# Book Reviews

**Published:** 1915-08

**Authors:** 


### Book Reviews.

HEREDITY AND EUGENICS. By various authors—Drs. Castle, Coulter, Davenport, East and Town. The University of Chicago Press. Price, $2.50.

Now that biology is firmly fixed as the science that teaches the improvement of animal and vegetable life, and the development of the human race, there is a growing demand on the part of the general reader for a knowledge of the fundamental principles that underlie this study. In this volume of three hundred pages, one gets the salient points regarding the laws of heredity, variation and evolution. He is shown how the germinal cell undergoes its modifications to form new variations; how these principles are applied to plant breeding and the inheritance of mental and physical characters. He is inducted into some of the mysteries of heredity and sex and the physical basis of evolution. Then follows a general application of all this knowledge to the betterment of the race.

We unreservedly recommend this book as being in every way worthy of its purpose and as a timely contribution towards the instruction of the general public.

MALONE DUGGAN.

---

The ninth volume of the Practical Medical Series of the Chicago Year-Book Publishers is upon the treatment of dermatoses by Baum and Moyer and Mitchell. It is a nicely bound volume of 220 pages; 166 pages on skin and venereal diseases, and the remainder miscellaneous papers by Dr. H. N. Moyer, many of which are interesting extracts from monographs on various medical subjects. Dr. Hazen says "mulattoes are more subject to skin diseases than are the blacks," and that dermatitis, keloids, syphilis, seborrhea, tenia, urticaria, vitiligo are the most common infections." Drug eruptions in epilepsy are usually due to the bromine compounds. A sudden skin eruption of any kind without prodromal symptoms may be a drug eruption. Iodides and bromides cause the most drug eruptions.

J. D. Barng "suggests that in a childless couple" we should examine the semen of the male before we curette the woman, as the fault lays with the man as often as with the woman from orchitis and other testicular inflammation.

Powell "recommends five feet of hydrostatic pressure and five pints of a solution from two to five thousand of permanganate at 98° F. in a gonorrhea. Blount valentine paint is recommended, as are daily irrigations. Baum recommends from two to three intravenous injections of Salvarsan, 0.4 to 0.6 grains, and from eight to twelve injections of calomel (10 per cent emulsion in syphilis). Reappearance of a positive Wassermann reaction should call for a repeated dose in which the neosalvarsan may be used.

Many other useful suggestions are found in the book.

